# Paraoxonase 1 polymorphism Q192R affects the pro-inflammatory cytokine TNF-alpha in healthy males

**DOI:** 10.1186/1756-0500-4-141

**Published:** 2011-05-10

**Authors:** Kai Lüersen, Constance Schmelzer, Christine Boesch-Saadatmandi, Christine Kohl, Gerald Rimbach, Frank Döring

**Affiliations:** 1 Institute of Human Nutrition and Food Science, Molecular Prevention, Christian-Albrechts-University of Kiel, Heinrich-Hecht-Platz 10, 24118 Kiel, Germany; 2 Institute of Human Nutrition and Food Science, Food Science, Christian-Albrechts-University of Kiel, Hermann-Rodewald-Straße 6, 24118 Kiel, Germany

## Abstract

**Background:**

Human paraoxonase 1 (PON1) is an HDL-associated enzyme with anti-oxidant/anti-inflammatory properties that has been suggested to play an important protective role against coronary heart diseases and underlying atherogenesis. The common *PON1 *Q192R polymorphism (*rs662*, A>G), a glutamine to arginine substitution at amino acid residue 192, has been analyzed in numerous association studies as a genetic marker for coronary heart diseases, however, with controversial results.

**Findings:**

To get a better understanding about the pathophysiological function of PON1, we analyzed the relationships between the Q192R polymorphism, serum paraoxonase activity and serum biomarkers important for atherogenesis. Genotyping a cohort of 49 healthy German males for the Q192R polymorphism revealed an allele distribution of 0.74 and 0.26 for the Q and R allele, respectively, typical for Caucasian populations. Presence of the R192 allele was found to be associated with a significantly increased paraoxonase enzyme activity of 187.8 ± 11.4 U/l in comparison to the QQ192 genotype with 60.5 ± 4.9 U/l. No significant differences among the genotypes were found for blood pressure, asymmetric dimethylarginine, LDL, HDL, triglycerides, and cholesterol. As expected, MIP-2 alpha a cytokine rather not related to atherosclerosis is not affected by the *PON1 *polymorphism. In contrast to that, the pro-inflammatory cytokine TNF-alpha is enhanced in R192 carriers (163.8 ± 24.7 pg/ml vs 94.7 ± 3.2 pg/ml in QQ192 carriers).

**Conclusions:**

Our findings support the hypothesis that the common *PON1 *R192 allele may be a genetic risk factor for atherogenesis by inducing chronic low-grade inflammation.

## Introduction

Paraoxonase 1 (PON1) is a calcium-dependent enzyme exhibiting esterase, lactonase and peroxidase activity. It accepts a broad range of substrates including organophosphates, diverse lactones and lipid peroxides and has been studied for its ability to breakdown pesticides and nerve gases. PON1 is a glycoprotein of about 45 kDa that is predominantly synthesized by the liver, from where it is distributed to other tissues, mainly to serum [1,2]. In serum, PON1 is associated with high-density-lipoprotein (HDL) particles [3]. HDL-associated PON1 has been frequently shown to have anti-oxidant and anti-inflammatory potential mainly by protecting lipids of HDLs and low-density lipoproteins (LDL) from oxidative modifications [1,2]. Most likely, these protective effects depend on the peroxidase and esterase activity of PON1 allowing the detoxification of oxidized molecules such as phospholipids and lipid hydroperoxides [4,5]. Cardiovascular diseases and underlying atherosclerosis are associated with oxidative stress and inflammation. Hence, serum PON1 is suggested to contribute to the established anti-atherogenic function of HDLs which is, at least partly, attributable to their anti-oxidative properties [1,2,6].

This notion is further supported by animal model studies using *PON1 *knock out and transgenic PON1 overexpressing mice. HDLs of *PON1*^-/- ^knock-out mice were found to prevent LDL oxidation less efficient than LDLs from control mice [7]. On the other hand, increased PON1 content in transgenic mice overexpressing murine or human PON1 resulted in HDLs that were more protected from lipid peroxidation [8,9]. Moreover, *PON1 *deficiency in mice resulted in elevated levels of oxidative stress and endothelial adhesion molecules [10]. Accordingly, *PON1*^-/- ^animals exhibited increased susceptibility to the development of large atherosclerotic lesions on a high-fat diet [7], whereas mice overexpressing human PON1 exhibited decreased atherosclerotic lesion sizes when fed an atherogenic diet [8,9].

The human *PON1 *gene is located on the long arm of chromosome 7 between q21 and q22. Two common coding region polymorphisms occur: a glutamine to arginine substitution at position 192 (Q192R) which affects PON1 enzyme activity and is analyzed in this study, and a leucine to methionine substitution at position 55 (L55M) [11]. *PON1 *gene polymorphisms have been examined with respect to their association to various human diseases including coronary heart disease (CHD), Parkinson's disease, type 2 diabetes and inflammatory bowel disease [12,13]. Most studies focused on the anti-oxidant/anti-inflammatory properties of PON1 in association with the development of atherosclerosis and the role of the Q192R polymorphism as a genetic marker for CHD. However, the results reported so far are controversial, some indicating an association between the Q192R polymorphism and atherosclerosis and CHD risk, while others do not as reviewed in [12,13].

In the present study, we have analyzed the influence of the *PON1 *Q192R polymorphism on serum lipids and inflammatory biomarkers in a cohort of 49 healthy male individuals to get a better understanding of the role of the *PON1 *Q192R polymorphism in the development of atherosclerosis and CHD.

## Materials and methods

### Participants and study design

The cohort of 53 healthy males investigated in the present study has been recently described [14]. In short, based on clinical laboratory tests, the participants aged between 21 and 48 had an average Body Mass Index (BMI) of 24.1 ± 2.5 and fulfilled four criteria: (i) no history of gastrointestinal, hepatic, cardiovascular or renal disease, (ii) no supplemental vitamin use for ≥ 2 weeks before the start of the study, (iii) non- or occasional smoking (≤ 3 cigarettes/day), and (iv) perpetuation of usual nutrition habits. Fasting blood samples were taken from each participant for genotyping, PON1 enzyme activity determination and inflammatory biomarker analyses. The study was approved by the ethics committee of the Medical Faculty of Kiel University, Germany, (permission number A121/07) and was conformed to Helsinki Declaration. All volunteers gave written informed consent prior to participation.

### Genotyping

Genomic DNA was isolated from whole blood samples. Genotyping of the *PON1 *Single Nucleotide Polymorphism (SNP) A/A, A/G, G/G (rs662) responsible for the Q192R substitutions was performed by using the TaqMan system. Fluorescence was measured with ABI Prism 7900 HT sequence detection system (ABI, Foster City, USA).

### PON enzyme activity assay

PON enzyme activity was determined spectrophotometrically in plasma samples following the protocol described in [15]. Briefly, the rate of hydrolysis of paraoxon (diethyl-p-nitrophenyl phosphatate; Supelco) was measured by monitoring the increase of absorbance at 405 nm using 100 mM Tris-HCl (pH 8.0), 1 mM paraoxon and 2 mM CaCl_2_. One unit of PON activity is defined as 1 nmol of 4-nitrophenol formed per minute at 20 °C under standard assay conditions (ε = 17600 M^-1 ^cm^-1^).

### Serum biomarkers

Supernatants of whole blood samples were measured with commercially available ELISA kits for TNF-alpha, MCP-1 (R&D Systems, Minneapolis, MN), oxLDL (KAMIYA Biomedical Company, Seattle, USA), asymmetric dimethylarginine (ADMA) (DLD Diagnostika, Hamburg, Germany) and MIP-2-alpha (Promocell, Heidelberg, Germany). Optical density was read on a microplate reader (Spectramax^® ^190, Molecular Devices). Laboratory measurements including serum lipid concentrations have been described previously [16].

### Statistical analysis

Results are displayed as means ± SEM. Data were analyzed by an unpaired two-sided Student's t-test (Microsoft Excel Version 2003 or GraphPad Prism 4.0 software). P-values < 0.05 were considered statistically significant.

## Results

### Genotype distribution and basic characteristics

Genotype analysis of the *PON1 *Q192R polymorphism (*rs662*, A>G) of 53 male volunteers revealed 25 homozygous for Q/Q (51%), 23 heterozygous for Q/R (47%) and 1 homozygous for R/R (2%), while 4 probes failed genotyping. Because of the small incidence, in further studies the G/G genotype (phenotype R/R) was combined with the A/G genotype (phenotype Q/R) group. Basic characteristics of the cohort have been recently reported by our group [14]. Briefly, the values for age (30.13 ± 6.71 years), weight (79.11 ± 10.17 kg), height (1.81 ± 0.06 m), BMI (24.12 ± 2.50 kg/m^2^) and fasting glucose level (86.47 ± 10.68 mg/dl) as well as for the kidney and liver parameters creatinine (1.05 ± 0.10 m/dl), aspartate aminotransferase (30.09 ± 8.67 U/l), glutamate pyruvate transaminase (37.79 ± 14.73 U/l) and γ-glutamyl transpeptidase (20.49 ± 10.36 U/l) were in accordance with the inclusion criteria of the study and show values within the physiological range for healthy men.

### Effect of the Q192R polymorphism on PON1 activity

**Figure 1:**
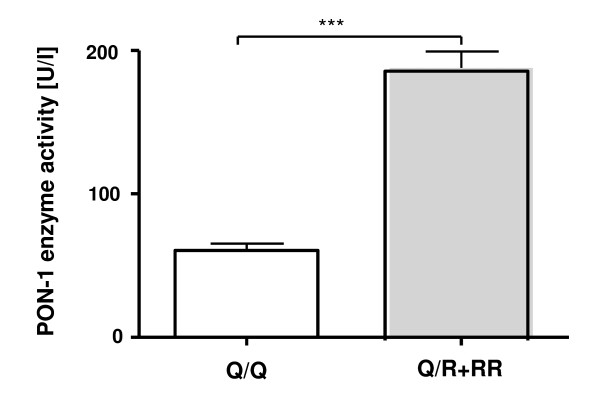
**Effect of the *PON1 *Q192R polymorphism on serum PON1 enzyme activity**. PON1 activity was determined in the sera of Q/Q (n = 25) and Q/R+R/R (n = 24) male individuals under standard assay conditions using paraoxon as substrate. Data represent means ± SEM (*** p ≤ 0.001).

PON1 enzyme activity was found to be significantly elevated in the serum of Q/R and R/R individuals with a mean value of 187.8 ± 11.4 U/l, when compared to the Q/Q group having a mean value of 60.5 ± 4.9 U/l (p < 0.001; unpaired two-sided Student's t-test) (Figure 1). These data confirm previous studies that have demonstrated a similar effect of the Q192R polymorphism on serum PON1 activity [4,15].

### Effect of the Q192R polymorphism on blood pressure and ADMA

Subsequently we analyzed a possible association between the Q192R polymorphism and blood pressure. However, no significant differences for blood pressure and ADMA levels, a blood pressure biomarker, were found between the Q/Q and the combined Q/R + R/R group with mean values of 82.5 ± 2.0 versus 82.9 ± 1.9 for diastolic and 125.0 ± 2.4 versus 128.0 ± 2.6 for systolic pressure as well as 0.96 ± 0.05 versus 0.98 ± 0.07 μmol/l for ADMA.

### Effect of the Q192R polymorphism on serum lipoproteins and lipids

The Q192R polymorphism had no significant effect on HDL (51.7 ± 2.8 versus 50.8 ± 2.4 mg/dl), LDL (95.8 ± 5.7 versus 92.8 ± 6.1 mg/dl), triglycerides (TG) (91.0 ± 8.1 versus 102.9 ± 11.6 mg/dl) and cholesterol (165.7 ± 5.1 versus 164.1 ± 6.7 mmol/l). In addition, we found a slight but not significant increase in the oxLDL levels for the Q/R + R/R group with a mean of 21.7 ± 2.8 U/ml compared to Q/Q individuals with a mean value of 17.8 ± 3.0 U/ml.

### Effect of the Q192R polymorphism on inflammatory biomarkers

**Figure 2:**
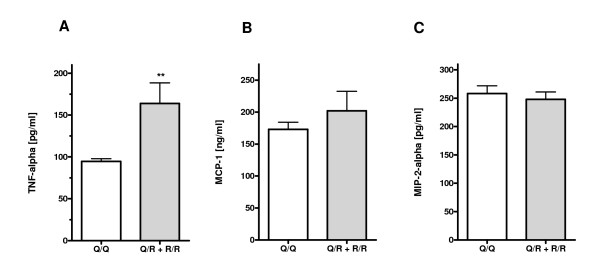
**Effect of the *PON1 *Q192R polymorphism on inflammatory biomarkers**. (A) TNF-alpha, (B) MCP-1 and (C) MIP-2-alpha levels were determined in the sera of Q/Q (n = 25) and Q/R+R/R (n = 24) male subjects. Data represent means ± SEM (** p ≤ 0.01).

Finally, we analyzed the effect of the Q192R polymorphism on the inflammatory biomarkers TNF-alpha, MCP-1 and MIP-2-alpha. As shown in Figure 2A, the mean level of TNF-alpha for the Q/Q group was found to be 94.7 ± 3.2 pg/ml. In the sera of Q/R and R/R individuals the respective level was significantly enhanced with a mean value of 163.8 ± 24.7 pg/ml (p = 0.007; unpaired two-sided Student's t-test). Furthermore, the inflammatory biomarker MCP-1 was slightly but not significantly enhanced in Q/R and R/R individuals when compared with the Q/Q group (202.0 ± 30.7 versus 173.0 ± 11.2 ng/ml) (Figure 2B). In contrast to that, MIP-2-alpha levels were not affected by the Q192R polymorphism (258.2 ± 13.7 versus 248.1 ± 12.9 pg/ml; Figure 2C).

## Discussion

The role of the *PON1 *Q192R polymorphism in cardiovascular diseases is still under debate [1,2,12,13]. In the present study, genotyping of a cohort of 49 German males revealed a frequency of 0.74 for the Q192 allele confirming previous reports on Western populations with Caucasian origin [17-20]. Moreover, we found that the occurrence of the R192 allele led to an elevated serum paraoxonase activity which is also in good accordance with previously published data on the Q and R allozymes [21,22]. In this regard it is noteworthy that paraoxon represents a non-natural substrate and that the natural substrate(s) of PON1 has/have not been identified so far [2]. Consequently, it is not a contradiction that elevated specific paraoxonase activities of PON1 allozymes have been demonstrated to be negatively correlated with their antioxidant capacity in HDLs, i.e. protecting LDLs against oxidation, reversing the biological effects of oxidised LDLs and preserving the function of HDLs [13,21,22]. Since the oxidation of LDL and the accompanied formation of foam cell layers are thought to represent crucial steps in the initiation process of atherosclerosis [23,24], an enhanced antioxidant activity of HDLs has been suggested to prevent atherosclerosis and CHD [1,2]. Accordingly, a low-active paraoxonase allele such as *PON1 *Q192 should protect against atherosclerosis when compared with the corresponding high-active R192 allele. Although such an association has been found in some studies [17-19,25-31], no relationship has been revealed by others [20,32-38]. Recent meta-analyses suggested a weak association between the *PON1 *Q192R polymorphism and CHD risk [39], however, no or only a population-specific effect of the R192 allele on human longevity [40]. Here we addressed the question whether the Q192R polymorphism and the related differences in PON1 activity are linked to changes in biomarkers indicative for a pro-atherogenic status.

Our analyses revealed that *PON1 *genotypes are not associated with alterations in blood pressure and ADMA levels, elevation of both are linked with atherosclerosis [41]. Moreover, there were no differences in the serum lipid profiles including TG, HDL, LDL and cholesterol, except for slightly but not significantly enhanced oxLDL levels. Similar results for TG, HDL, LDL and cholesterol have been reported previously [19,20,25,29,31-34,36-38], whereas a few studies found a more pro-atherogenic serum lipid and/or lipoprotein pattern in association with the R192 allele [35,42]. The principal finding of this study is that the frequency of the low-antioxidant R192 allele is associated with significantly increased levels of the pro-inflammatory cytokine TNF-alpha. Since chronic low grade alterations of inflammatory markers are known to be associated with increased atherogenic risk [43], elevated R192 allele-dependent TNF-alpha levels may thus represent a putative risk marker.

Only recently, it has been demonstrated that adenovirus-based overexpression of human PON1 in apolipoprotein E knock-out mice caused enhanced serum anti-oxidative and anti-inflammatory capabilities reflected among other factors by decreased TNF-alpha levels [44]. Hence, enhanced PON1 antioxidant capacity was found to be associated with reduced TNF-alpha levels, probably protective against atherosclerosis. Considering the anti-oxidant property of HDL-associated PON1, it is intriguing to speculate that low amounts of serum PON1 or low-anti-oxidant *PON1 *alleles such as R192 lead to elevated levels of reactive oxygen species in this way triggering the redox-sensitive NfkappaB signaling pathway that is known to stimulate TNF-alpha expression. However, further mechanistic investigations are necessary to decipher this proposed PON1 TNF-alpha relationship. It is remarkable that, in turn, enhanced TNF-alpha levels have been shown to down-regulate PON1 expression in murine and human hepatoma cell lines as well as *in vivo *in mice, most probably via an NF-kappaB- and nuclear receptor peroxisome proliferator-activated receptor-alpha (PPAR-alpha)-dependent mechanism thereby diminishing the antioxidant and anti-atherogenic activity of HDLs [45,46]. Moreover, a TNF-alpha antagonist therapy in rheumatoid arthritis patients led to enhanced PON1 levels concurrent with elevated anti-oxidative capacities of HDLs and lowered inflammatory status [47]. Interestingly, anti-TNF-alpha therapy response was found to be associated with SNPs in the *PON1 *locus [48] and a recent case-control study on atherosclerosis in rheumatoid arthritis found a correlation between PON-1 activity and serum TNF-alpha and IL-6 levels [49], emphasizing the close regulatory interrelation between PON1 activity and TNF-alpha levels.

In addition to TNF alpha, we found that levels of a second pro-inflammatory cytokine MCP-1 were slightly although not significantly enhanced in R192 carriers that usually express PON1 allozymes with less antioxidant capacity. MCP-1 expression and secretion of endothelial cells are known to be induced by oxidized LDL and accordingly, are thought to represent a crucial step in the initial phase of the inflammatory processes in atherosclerosis [43]. Consistent with its anti-oxidant function that leads to reduced lipid peroxidation, PON1 has been demonstrated to attenuate MCP-1 expression of cultured endothelial cells [50]. In good accordance with that are data on HDLs isolated from wild type and *PON1 *knock-out mice. In a human endothelial cell culture model *PON1^-/- ^*HDLs exhibited significantly less antioxidant capacity accompanied with elevated MCP-1 levels, hence, linking *PON1 *deficiency to lipid hydroperoxide-triggered expression of pro-inflammatory MCP-1 [7]. Since the amount of MIP-2 alpha, a cytokine rather not related to atherosclerosis, is not affected by *PON1 *polymorphism, the observed changes of TNF-alpha and MCP-1 levels observed in the current study are most likely specific.

## Conclusion

Our data indicate that the low-antioxidant *PON1 *R192 allele is associated with increased pro-inflammatory cytokines known to be involved in the initiation process of atherosclerosis. However, our finding needs to be confirmed in further studies and especially in larger study population.

## Abbreviations used

ADMA: asymmetric dimethylarginine; CHD: coronary heart disease; PON1: paraoxonase 1; SNP: single nucleotide polymorphism; TG: triglycerides.

## Competing interests

The authors declare that they have no competing interests.

## Authors' contributions

KL analysed the data and wrote the manuscript. CS participated in the design of the study, acquired and analysed data. CBS performed enzyme assays. CK carried out genotyping studies. GR participates in the design of the study and critically revised the manuscript. FD conceived and designed the study, analysed the data and wrote the manuscript. All authors read and approved the final manuscript.

## References

[B1] Ng CJ Shih DM Hama SY Villa N Navab M Reddy ST The paraoxonase gene family and atherosclerosis Free Radic Biol Med 2005 38 2 153 163 10.1016/j.freeradbiomed.2004.09.035 15607899

[B2] Precourt LP Amre D Denis MC Lavoie JC Delvin E Seidman E Levy E The three-gene paraoxonase family: Physiologic roles, actions and regulation Atherosclerosis 2011 214 1 20 36 10.1016/j.atherosclerosis.2010.08.076 20934178

[B3] Mackness MI Hallam SD Peard T Warner S Walker CH The separation of sheep and human serum "A"-esterase activity into the lipoprotein fraction by ultracentrifugation Comp Biochem Physiol B 1985 82 4 675 677 10.1016/0305-0491(85)90506-1 3004805

[B4] Aviram M Hardak E Vaya J Mahmood S Milo S Hoffman A Billicke S Draganov D Rosenblat M Human serum paraoxonases (PON1) Q and R selectively decrease lipid peroxides in human coronary and carotid atherosclerotic lesions: PON1 esterase and peroxidase-like activities Circulation 2000 101 21 2510 2517 10831526 10.1161/01.cir.101.21.2510

[B5] Watson AD Berliner JA Hama SY La Du BN Faull KF Fogelman AM Navab M Protective effect of high density lipoprotein associated paraoxonase. Inhibition of the biological activity of minimally oxidized low density lipoprotein J Clin Invest 1995 96 6 2882 2891 10.1172/JCI118359 8675659 PMC185999

[B6] Soran H Younis NN Charlton-Menys V Durrington P Variation in paraoxonase-1 activity and atherosclerosis Curr Opin Lipidol 2009 20 4 265 274 10.1097/MOL.0b013e32832ec141 19550323

[B7] Shih DM Gu L Xia YR Navab M Li WF Hama S Castellani LW Furlong CE Costa LG Fogelman AM Mice lacking serum paraoxonase are susceptible to organophosphate toxicity and atherosclerosis Nature 1998 394 6690 284 287 10.1038/28406 9685159

[B8] Oda MN Bielicki JK Ho TT Berger T Rubin EM Forte TM Paraoxonase 1 overexpression in mice and its effect on high-density lipoproteins Biochem Biophys Res Commun 2002 290 3 921 927 10.1006/bbrc.2001.6295 11798161

[B9] Tward A Xia YR Wang XP Shi YS Park C Castellani LW Lusis AJ Shih DM Decreased atherosclerotic lesion formation in human serum paraoxonase transgenic mice Circulation 2002 106 4 484 490 10.1161/01.CIR.0000023623.87083.4F 12135950

[B10] Ng DS Chu T Esposito B Hui P Connelly PW Gross PL Paraoxonase-1 deficiency in mice predisposes to vascular inflammation, oxidative stress, and thrombogenicity in the absence of hyperlipidemia Cardiovasc Pathol 2008 17 4 226 232 10.1016/j.carpath.2007.10.001 18402813

[B11] Humbert R Adler DA Disteche CM Hassett C Omiecinski CJ Furlong CE The molecular basis of the human serum paraoxonase activity polymorphism Nat Genet 1993 3 1 73 76 10.1038/ng0193-73 8098250

[B12] Furlong CE Suzuki SM Stevens RC Marsillach J Richter RJ Jarvik GP Checkoway H Samii A Costa LG Griffith A Human PON1, a biomarker of risk of disease and exposure Chem Biol Interact 187 1-3 355 361 10.1016/j.cbi.2010.03.033 PMC3035622 20338154

[B13] Li HL Liu DP Liang CC Paraoxonase gene polymorphisms, oxidative stress, and diseases J Mol Med 2003 81 12 766 779 10.1007/s00109-003-0481-4 14551701

[B14] Schmelzer C Niklowitz P Okun JG Haas D Menke T Döring F Ubiquinol-induced gene expression signatures are translated into reduced erythropoiesis and LDL cholesterol levels in humans IUBMB Life 2011 63 1 42 48 10.1002/iub.413 21280176

[B15] Boesch-Saadatmandi C Rimbach G Schrader C Kofler BM Armah CK Minihane AM Determinants of paraoxonase activity in healthy adults Mol Nutr Food Res 54 12 1842 1850 10.1002/mnfr.201000190 20658496

[B16] Egert S Wolffram S Bosy-Westphal A Boesch-Saadatmandi C Wagner AE Frank J Rimbach G Mueller MJ Daily quercetin supplementation dose-dependently increases plasma quercetin concentrations in healthy humans J Nutr 2008 138 9 1615 1621 18716159 10.1093/jn/138.9.1615

[B17] Ruiz J Blanche H James RW Garin MC Vaisse C Charpentier G Cohen N Morabia A Passa P Froguel P Gln-Arg192 polymorphism of paraoxonase and coronary heart disease in type 2 diabetes Lancet 1995 346 8979 869 872 10.1016/S0140-6736(95)92709-3 7564671

[B18] Serrato M Marian AJ A variant of human paraoxonase/arylesterase (HUMPONA) gene is a risk factor for coronary artery disease J Clin Invest 1995 96 6 3005 3008 10.1172/JCI118373 8675673 PMC186013

[B19] Regieli JJ Jukema JW Doevendans PA Zwinderman AH Kastelein JJ Grobbee DE van der Graaf Y Paraoxonase variants relate to 10-year risk in coronary artery disease: impact of a high-density lipoprotein-bound antioxidant in secondary prevention J Am Coll Cardiol 2009 54 14 1238 1245 10.1016/j.jacc.2009.05.061 19778663

[B20] Turban S Fuentes F Ferlic L Brugada R Gotto AM Ballantyne CM Marian AJ A prospective study of paraoxonase gene Q/R192 polymorphism and severity, progression and regression of coronary atherosclerosis, plasma lipid levels, clinical events and response to fluvastatin Atherosclerosis 2001 154 3 633 640 10.1016/S0021-9150(00)00495-0 11257264

[B21] Mackness B Mackness MI Arrol S Turkie W Durrington PN Effect of the human serum paraoxonase 55 and 192 genetic polymorphisms on the protection by high density lipoprotein against low density lipoprotein oxidative modification FEBS Lett 1998 423 1 57 60 10.1016/S0014-5793(98)00064-7 9506841

[B22] Aviram M Billecke S Sorenson R Bisgaier C Newton R Rosenblat M Erogul J Hsu C Dunlop C La Du B Paraoxonase active site required for protection against LDL oxidation involves its free sulfhydryl group and is different from that required for its arylesterase/paraoxonase activities: selective action of human paraoxonase allozymes Q and R Arterioscler Thromb Vasc Biol 1998 18 10 1617 1624 9763535 10.1161/01.atv.18.10.1617

[B23] Yla-Herttuala S Palinski W Rosenfeld ME Parthasarathy S Carew TE Butler S Witztum JL Steinberg D Evidence for the presence of oxidatively modified low density lipoprotein in atherosclerotic lesions of rabbit and man J Clin Invest 1989 84 4 1086 1095 10.1172/JCI114271 2794046 PMC329764

[B24] Steinberg D Parthasarathy S Carew TE Khoo JC Witztum JL Beyond cholesterol. Modifications of low-density lipoprotein that increase its atherogenicity N Engl J Med 1989 320 14 915 924 10.1056/NEJM198904063201407 2648148

[B25] Sanghera DK Saha N Aston CE Kamboh MI Genetic polymorphism of paraoxonase and the risk of coronary heart disease Arterioscler Thromb Vasc Biol 1997 17 6 1067 1073 9194756 10.1161/01.atv.17.6.1067

[B26] Tobin MD Braund PS Burton PR Thompson JR Steeds R Channer K Cheng S Lindpaintner K Samani NJ Genotypes and haplotypes predisposing to myocardial infarction: a multilocus case-control study Eur Heart J 2004 25 6 459 467 10.1016/j.ehj.2003.11.014 15039125

[B27] Lakshmy R Ahmad D Abraham RA Sharma M Vemparala K Das S Reddy KS Prabhakaran D Paraoxonase gene Q192R & L55M polymorphisms in Indians with acute myocardial infarction & association with oxidized low density lipoprotein Indian J Med Res 131 522 529 20424303

[B28] Gluba A Pietrucha T Banach M Piotrowski G Rysz J The role of polymorphisms within paraoxonases (192 Gln/Arg in PON1 and 311Ser/Cys in PON2) in the modulation of cardiovascular risk: a pilot study Angiology 61 2 157 165 10.1177/0003319709351258 19939821

[B29] Odawara M Tachi Y Yamashita K Paraoxonase polymorphism (Gln192-Arg) is associated with coronary heart disease in Japanese noninsulin-dependent diabetes mellitus J Clin Endocrinol Metab 1997 82 7 2257 2260 10.1210/jc.82.7.2257 9215303

[B30] Zama T Murata M Matsubara Y Kawano K Aoki N Yoshino H Watanabe G Ishikawa K Ikeda Y A 192Arg variant of the human paraoxonase (HUMPONA) gene polymorphism is associated with an increased risk for coronary artery disease in the Japanese Arterioscler Thromb Vasc Biol 1997 17 12 3565 3569 9437206 10.1161/01.atv.17.12.3565

[B31] Imai Y Morita H Kurihara H Sugiyama T Kato N Ebihara A Hamada C Kurihara Y Shindo T Oh-hashi Y Evidence for association between paraoxonase gene polymorphisms and atherosclerotic diseases Atherosclerosis 2000 149 2 435 442 10.1016/S0021-9150(99)00340-8 10729395

[B32] Antikainen M Murtomaki S Syvanne M Pahlman R Tahvanainen E Jauhiainen M Frick MH Ehnholm C The Gln-Arg191 polymorphism of the human paraoxonase gene (HUMPONA) is not associated with the risk of coronary artery disease in Finns J Clin Invest 1996 98 4 883 885 10.1172/JCI118869 8770857 PMC507500

[B33] Rice GI Ossei-Gerning N Stickland MH Grant PJ The paraoxonase Gln-Arg 192 polymorphism in subjects with ischaemic heart disease Coron Artery Dis 1997 8 11-12 677 682 9472455 10.1097/00019501-199711000-00001

[B34] Herrmann SM Blanc H Poirier O Arveiler D Luc G Evans A Marques-Vidal P Bard JM Cambien F The Gln/Arg polymorphism of human paraoxonase (PON 192) is not related to myocardial infarction in the ECTIM Study Atherosclerosis 1996 126 2 299 303 10.1016/0021-9150(96)05917-5 8902155

[B35] Ombres D Pannitteri G Montali A Candeloro A Seccareccia F Campagna F Cantini R Campa PP Ricci G Arca M The gln-Arg192 polymorphism of human paraoxonase gene is not associated with coronary artery disease in italian patients Arterioscler Thromb Vasc Biol 1998 18 10 1611 1616 9763534 10.1161/01.atv.18.10.1611

[B36] Ko YL Ko YS Wang SM Hsu LA Chang CJ Chu PH Cheng NJ Chen WJ Chiang CW Lee YS The Gln-Arg 191 polymorphism of the human paraoxonase gene is not associated with the risk of coronary artery disease among Chinese in Taiwan Atherosclerosis 1998 141 2 259 264 10.1016/S0021-9150(98)00179-8 9862174

[B37] Suehiro T Nakauchi Y Yamamoto M Arii K Itoh H Hamashige N Hashimoto K Paraoxonase gene polymorphism in Japanese subjects with coronary heart disease Int J Cardiol 1996 57 1 69 73 10.1016/S0167-5273(96)02779-9 8960946

[B38] Aubo C Senti M Marrugat J Tomas M Vila J Sala J Masia R Risk of myocardial infarction associated with Gln/Arg 192 polymorphism in the human paraoxonase gene and diabetes mellitus. The REGICOR Investigators Eur Heart J 2000 21 1 33 38 10.1053/euhj.1999.1660 10610741

[B39] Wang M Lang X Zou L Huang S Xu Z Four genetic polymorphisms of paraoxonase gene and risk of coronary heart disease: a meta-analysis based on 88 case-control studies Atherosclerosis 214 2 377 385 10.1016/j.atherosclerosis.2010.11.028 21146823

[B40] Caliebe A Kleindorp R Blanche H Christiansen L Puca AA Rea IM Slagboom E Flachsbart F Christensen K Rimbach G No or only population-specific effect of PON1 on human longevity: a comprehensive meta-analysis Ageing Res Rev 2010 9 3 238 244 10.1016/j.arr.2010.03.003 20362697

[B41] Boger RH Asymmetric dimethylarginine, an endogenous inhibitor of nitric oxide synthase, explains the "L-arginine paradox" and acts as a novel cardiovascular risk factor J Nutr 2004 134 10 Suppl 2842S 2847S discussion 2853S 15465797 10.1093/jn/134.10.2842S

[B42] Hegele RA Brunt JH Connelly PW A polymorphism of the paraoxonase gene associated with variation in plasma lipoproteins in a genetic isolate Arterioscler Thromb Vasc Biol 1995 15 1 89 95 7749820 10.1161/01.atv.15.1.89

[B43] Braunersreuther V Mach F Steffens S The specific role of chemokines in atherosclerosis Thromb Haemost 2007 97 5 714 721 17479181

[B44] Zhang C Peng W Wang M Zhu J Zang Y Shi W Zhang J Qin J Studies on protective effects of human paraoxonases 1 and 3 on atherosclerosis in apolipoprotein E knockout mice Gene Ther 17 5 626 633 10.1038/gt.2010.11 20182519

[B45] Kumon Y Suehiro T Ikeda Y Hashimoto K Human paraoxonase-1 gene expression by HepG2 cells is downregulated by interleukin-1beta and tumor necrosis factor-alpha, but is upregulated by interleukin-6 Life Sci 2003 73 22 2807 2815 10.1016/S0024-3205(03)00704-5 14511766

[B46] Han CY Chiba T Campbell JS Fausto N Chaisson M Orasanu G Plutzky J Chait A Reciprocal and coordinate regulation of serum amyloid A versus apolipoprotein A-I and paraoxonase-1 by inflammation in murine hepatocytes Arterioscler Thromb Vasc Biol 2006 26 8 1806 1813 10.1161/01.ATV.0000227472.70734.ad 16709944

[B47] Popa C van Tits LJ Barrera P Lemmers HL van den Hoogen FH van Riel PL Radstake TR Netea MG Roest M Stalenhoef AF Anti-inflammatory therapy with tumour necrosis factor alpha inhibitors improves high-density lipoprotein cholesterol antioxidative capacity in rheumatoid arthritis patients Ann Rheum Dis 2009 68 6 868 872 10.1136/ard.2008.092171 18635596

[B48] Liu C Batliwalla F Li W Lee A Roubenoff R Beckman E Khalili H Damle A Kern M Furie R Genome-wide association scan identifies candidate polymorphisms associated with differential response to anti-TNF treatment in rheumatoid arthritis Mol Med 2008 14 9-10 575 581 18615156 10.2119/2008-00056.Liu PMC2276142

[B49] Kerekes G Szekanecz Z Der H Sandor Z Lakos G Muszbek L Csipo I Sipka S Seres I Paragh G Endothelial dysfunction and atherosclerosis in rheumatoid arthritis: a multiparametric analysis using imaging techniques and laboratory markers of inflammation and autoimmunity J Rheumatol 2008 35 3 398 406 18203326

[B50] Mackness B Hine D Liu Y Mastorikou M Mackness M Paraoxonase-1 inhibits oxidised LDL-induced MCP-1 production by endothelial cells Biochem Biophys Res Commun 2004 318 3 680 683 10.1016/j.bbrc.2004.04.056 15144891

